# Culture-Dependent and Amplicon Sequencing Approaches Reveal Diversity and Distribution of Black Fungi in Antarctic Cryptoendolithic Communities

**DOI:** 10.3390/jof7030213

**Published:** 2021-03-16

**Authors:** Laura Selbmann, Gerardo A. Stoppiello, Silvano Onofri, Jason E. Stajich, Claudia Coleine

**Affiliations:** 1 Department of Ecological and Biological Sciences, University of Tuscia, 01100 Viterbo, Italy; selbmann@unitus.it (L.S.); stoppiello@unitus.it (G.A.S.); onofri@unitus.it (S.O.); 2 Mycological Section, Italian Antarctic National Museum (MNA), 16121 Genoa, Italy; 3 Department of Microbiology and Plant Pathology, University of California, Riverside, CA 92521, USA

**Keywords:** Antarctica, cryptoendolithic communities, metabarcoding, black fungi, extremophiles

## Abstract

In the harshest environmental conditions of the Antarctic desert, normally incompatible with active life, microbes are adapted to exploit the cryptoendolithic habitat (i.e., pore spaces of rocks) and represent the predominant life-forms. In the rocky niche, microbes take advantage of the thermal buffering, physical stability, protection against UV radiation, excessive solar radiation, and water retention—of paramount importance in one of the driest environments on Earth. In this work, high-throughput sequencing and culture-dependent approaches have been combined, for the first time, to untangle the diversity and distribution of black fungi in the Antarctic cryptoendolithic microbial communities, hosting some of the most extreme-tolerant microorganisms. Rock samples were collected in a vast area, along an altitudinal gradient and opposite sun exposure—known to influence microbial diversity—with the aim to compare and integrate results gained with the two approaches. Among black fungi, *Friedmanniomyces endolithicus* was confirmed as the most abundant taxon. Despite the much stronger power of the high-throughput sequencing, several species were not retrieved with DNA sequencing and were detectable by cultivation only. We conclude that both culture-dependent and -independent analyses are needed for a complete overview of black fungi diversity. The reason why some species remain undetectable with molecular methods are speculated upon. The effect of environmental parameters such as sun exposure on relative abundance was clearer if based on the wider biodiversity detected with the molecular approach.

## 1. Introduction

The cryptoendolithic microbial communities (e.g., microorganisms dwelling inside porous rocks) are successful ecosystems spreading in the Antarctic ice-free deserts, one of the harshest environments on Earth. In the Victoria Land, the McMurdo Dry Valleys are the largest of these areas, while additional scatterings of exposed naked rocks are represented by peaks of the Transantarctic Mountains, emerging from the Polar Plateau. Due to their prohibitive conditions, i.e., low temperatures, high and fast thermal excursions, low water availability, extreme dryness, and high incidence of solar and especially ultraviolet radiation (UV), the Dry Valleys are universally accounted as the best terrestrial analogue for the Martian environment. These harsh conditions, especially at high altitude, are incompatible with active life, and the endolithic lifestyle represents the last possibility for survival [1,2]. The endolithic niche, in fact, provides advantages such as protection from UV, from freeze–thaw events due to the thermal inertia of rocks, the physical stability of the substratum, and water retention [3,4].

Antarctic cryptoendolithic communities colonize quarzitic sandstone as the preferential substratum due to its homogeneous porosity and high translucence [5,6] and are typically organized in a colored and biologically distinct stratification at a depth of about 10 mm below the rock surface, according to the physiological requirements. Non-lichenized endemic chlorophyte algae and cyanobacteria develop in the deeper green band, where harmful solar radiation is more efficiently screened; immediately above, in the white band, lichenized fungi and algae develop loose but obligate symbiotic associations; both lichenized algae in the white band together with phototrophs in the green band and cyanobacteria act as primary producers, while other fungi and bacteria play a role as consumers. Highly melanized meristematic fungi are conspicuously present and play a fundamental protective role, screening excessive harmful solar radiation by forming a black barrier above the stratification of other biological compartments [7,8].

Black meristematic fungi are morphologically rather homogeneous but phylogenetically quite diverse, sharing the common ability to spread in the extremes [9,10]. These organisms are of special interest for their stunning capacity to withstand practically any kind of stress, including high ionizing radiation, space, and even simulated Mars conditions [11,12]. In particular, the terms meristematic, microcolonial (MCF), or rock-inhabiting fungi (RIF) specifically indicate black meristematic fungi perfectly adapted to life on and in the rocks [13,14,15,16,17].

Studies of these microbial communities have characterized biodiversity, composition, and responses to stresses. Deeper insight of these communities can inform models to predict the fate of border ecosystems on Earth under the present trajectory of climate change, the limits of adaptability for life and evolution in the extremes, as well as implications to speculate the possibility for life elsewhere in the universe [18,19]. The changes in biodiversity in response to increased environmental pressure has been widely investigated with molecular approaches, indicating that sun exposure plays a crucial role in the community composition taxon abundance and the distribution of functional groups of fungi [20,21,22], influencing these ecosystems more than other abiotic parameters (e.g., altitude and sea distance). Some RIF species, in particular, occurred with highest frequency in the shady south-exposed rock surfaces [23] that, being much colder than north sun-exposed surfaces, may affect the possibility for biological activity [24]. A culture-dependent method has also been applied and led to the description of new taxa, some of which are unique to this habitat, underlying the strong effect of genetic/geographic isolation over a timescale of evolutionary significance and environmental constraints in promoting adaptive radiation [7,8,10,15,16].

The aim of this study was to combine, for the first time on these specimens, and with a special focus on RIF as keystone taxa for their protective role, the strength of high-throughput sequencing (HTS) with the traditional cultivation method. Despite lower sensitivity, cultivation may allow detection of species that are overlooked with molecular tools and offers the opportunity to develop biological resources for functional and genetic studies. Moreover, the integration of cultivation and HTS has allowed a complete survey of microbial diversity, uncovering species that were previously neglected or unknown to science, when applied in samples from different extreme environments (e.g., [25,26]).

This study was based on a wide sampling area, spanning Antarctic regions in the Southern (McMurdo Dry Valleys) and Northern Victoria Land. The sampling included seventeen localities over a distance of about 400 km along an altitudinal gradient from 910 to 3100 m above sea level (a.s.l.). Moreover, both north- and south-exposed surfaces were sampled to compare the influence of sunlight exposure and intensity on RIF diversity and distribution.

## 2. Materials and Methods

### 2.1. Sampling Area

**Figure 1:**
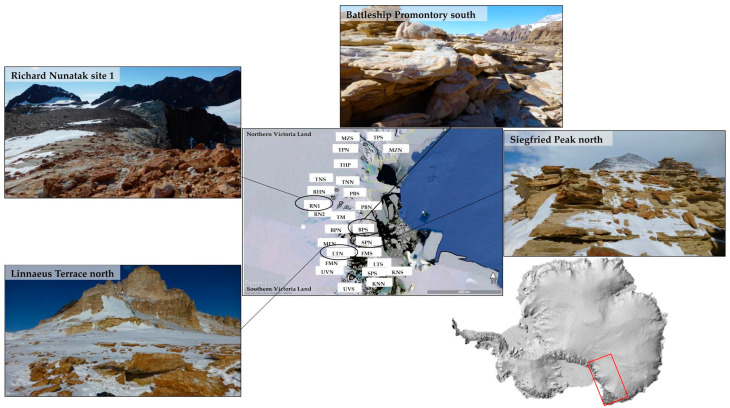
Map of the study area showing the location of the sampling sites spanning Northern and Southern Victoria Land (continental Antarctica). Sites correspondences are reported in Table S1. A map of Antarctica is included; the red square indicates the Victoria Land.

Sandstone rocks colonized by cryptoendolithic lichen-dominated communities were collected from fifteen localities along the Victoria Land (continental Antarctica) in a latitudinal transect ranging from 74°10′44.0″ S 162°30′53.0″ E (Mt. New Zealand, Northern Victoria Land) to 77°54′37.8″ S 161°34′48.8″ E (Knobhead, Southern Victoria Land) and from 910 (Battleship Promontory, Southern Victoria Land) to 3100 m a.s.l. (Mt. New Zealand, Northern Victoria Land) (Figure 1, Table S1). Both northern- and southern-exposed rock surfaces were sampled to compare the effect of sun exposure on black fungal diversity and distribution. White and red sandstones were sampled at Richard Nunatak.

Samples were collected during the XXXI Italian Antarctic Expedition (December 2015–January 2016) by Laura Selbmann. The presence of lithic colonization was first assessed by direct observation in situ using a magnifying lens. Rocks were excised using a geological hammer and sterile chisel, and samples were placed in sterile bags, transported, and stored at −20 °C in the Italian National Antarctic Museum’s Culture Collection of Fungi from Extreme Environments (MNA-CCFEE), hosted at the University of Tuscia (Italy), until downstream analysis was conducted.

### 2.2. Isolation and Identification of Culturable Fungi

For each location and sun exposure condition, two different rock samples were chosen and analyzed in duplicate; for one site (i.e., Richard Nunatak), two rock typologies were selected (white and red sandstones). A total of 191 plates were analyzed.

The rock fragments were then seeded on Petri dishes containing malt extract agar (MEA; malt extract 3%, agar 1.5%) (AppliChem GmbH, Darmstadt-Germany) with addition of 100 ppm chloramphenicol to inhibit bacterial growth. The plates were then incubated at 15 °C and colonies development was checked weekly. Colonies were then transferred onto sterile fresh medium (MEA) and incubated at 15 °C for a period of few weeks to a few months, until DNA extraction was conducted.

All the fungal strains obtained were cultivated on malt agar slants (MEA) and cryopreserved at −150 °C in the MNA-CCFEE, Mycological Section of the National Museum of Antarctica “Felice Ippolito”.

### 2.3. Identification of Fungal Strains

DNA was extracted from pure cultures using the Nucleospin Plant kit (Macherey-Nagel, Düren, Germany), according to the manufacturer’s instructions. The fungal internal transcribed spacer (ITS) was amplified using ITS5 (5′-GGAAGTAAAAGTCGTAACAAGG-3′) and ITS4 (5′-TCC TCCGCTTATTGATATGC-3′) primers. PCR reactions were performed using BioMix (BioLine GmbH, Luckenwalde, Germany). The PCR mixtures were prepared with 5 pmol of each primer and 20 ng of template DNA; Milli-Q sterile water was added to a final volume of 25 µL. Amplification was carried out using the MyCycler™ Thermal Cycler (Bio-Rad Laboratories, GmbH, Munich, Germany). PCR protocol was as follows: 3 min at 95 °C for the first denaturation step, then 35 cycles of a denaturation step at 95 °C for 30 s, an annealing step at 55 °C for 30 s, and an extension step at 72 °C for 30 s. The last extension was at 72 °C for 5 min. Amplicons were then purified using the Nucleospin Extract kit (Macherey-Nagel, Düren, Germany) and sequenced by Macrogen Inc. (Seoul, Korea). Sequence assembly was done using the software ChromasPro v1.32 (Technelysium, Southport, Queensland, Australia).

The ITS sequences were searched for matches in the National Center for Biotechnology Information (NCBI) database using the BLASTN program (https://blast.ncbi.nlm.nih.gov/Blast.cgi, accessed on 10 September 2020).

### 2.4. DNA Extraction and Metabarcoding Sequencing

The rock samples were crushed in sterile conditions using a hammer and chisel; metagenomic DNA was extracted from 1 g of powdered rocks using MOBIO Power Soil DNA Extraction kit (MOBIO Laboratories, Carlsbad, CA, USA). ITS1F (CTTGGTCATTTAGAGGAAGTAA) and ITS2 (GCTGCGTTCTTCATCGATGC) primers were used to amplify the ITS1 region for the fungal community according to Smith and Peay’s Illumina MiSeq protocol [27]. PCR reactions were performed in a total volume of 25 µL, containing 1 µL of each primer, 12.5 µL of Taq DNA Polymerase (Thermo Fisher Scientific Inc., Waltham, MA, USA), 9.5 µL of nuclease-free water (Sigma-Aldrich, St. Louis, MO, USA), and 5 ng of DNA. Amplification conditions were as above: initial denaturation at 93 °C for 3 min, 35 cycles of denaturation at 95 °C for 45 s, annealing at 50 °C for 1 min, and extension at 72 °C for 90 s, followed by a final extension at 72 °C for 10 min in an automated thermal cycler (Bio Rad, Hercules, CA, USA). Amplicons were purified using the NucleoSpin Gel PCR Clean-up kit (Macherey-Nagel, Hoerdt, France), quantified using the Qubit dsDNA HS Assay Kit (Life Technologies, Camarillo, CA, USA), and then barcoded and pooled to produce an equimolar mixture. Metabarcoding sequencing (paired-end reads, 2 × 300 bp) was performed on the Illumina Miseq platform at the Institute for Integrative Genome Biology, University of California, Riverside. Two replicates for each site were extracted, amplified, and sequenced; all replicates’ datasets were merged to increase the amount of sequence information.

Raw sequencing data have been archived in NCBI SRA under the accession number PRJNA453198.

### 2.5. Bioinformatics Analysis and Downstream Analysis

The ITS1 amplicon sequencing dataset was processed with AMPtk: Amplicon ToolKit for NGS data (formally UFITS) (https://github.com/nextgenusfs/amptk) v1.0.0 [28]. Briefly, barcodes and primers were removed from raw data. Reads were then subjected to quality trimming to a maximum of 300 bp and reads less than 200 bp in length were discarded, and chimera removal was performed utilizing USEARCH v9.1.13 with default parameters [29]. Sequence quality filtering was performed with the expected error parameter of 0.9 and the cleaned dataset was clustered with UPARSE using a 97% percent identity parameter to generate the operational taxonomic units (OTUs). Global singletons and rare taxa (<5 reads) were discarded as recommended by Lindahl et al. [30]. Finally, taxonomic identification was performed with the hybrid database SINTAX/UTAX [29].

As the main purpose was to focus on biodiversity and distribution of black fungi, ITS sequences belonging to this group (i.e., members of Capnodiales in the class Dothideomycetes and Chaetothyriales in the class Eurotiomycetes) were filtered from the whole ITS1 dataset and used for downstream analysis.

Upset and Venn plots were constructed to show the number of shared OTUs among north- and south-exposed samples using Intervene (https://intervene.shinyapps.io/intervene/) [31] and Venny (http://bioinfogp.cnb.csic.es/tools/venny) [32], respectively. Altitude values and species richness and abundance were correlated using MICtools v1.1.4. [33], that allowed us to identify the relationships of various degrees of complexity reporting the Pearson correlation coefficient, the Spearman rank coefficient, and the maximal information coefficient (MIC) [34].

## 3. Results

### 3.1. Occurrence of Black Fungi in the Antarctic Endolithic Communities through Culture-Dependent Approach and Amplicon Sequencing

**Figure 2:**
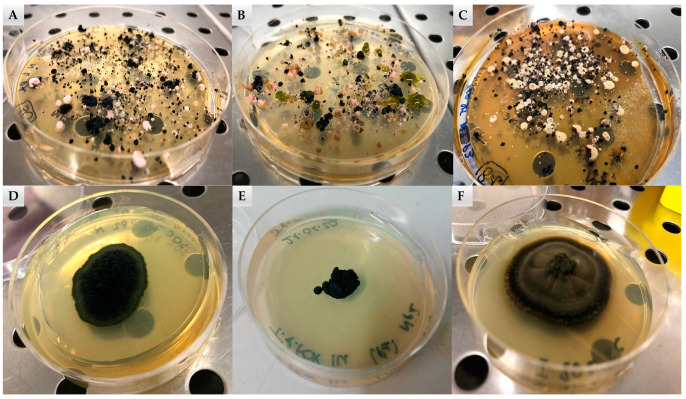
(**A**–**C**) Colonies grown on malt extract agar, isolated from crushed rocks. Linnaeus Terrace north (**A**), Mt. Elektra (**B**), and Finger Mountain (**C**). (**D**–**F**) Examples of black fungal colonies in pure cultures isolated from Antarctic endolithic communities: *Recurvomyces mirabilis* (**D**), *Friedmanniomyces endolithicus* (**E**), and *Extremus antarcticus* (**F**).

**Figure 3:**
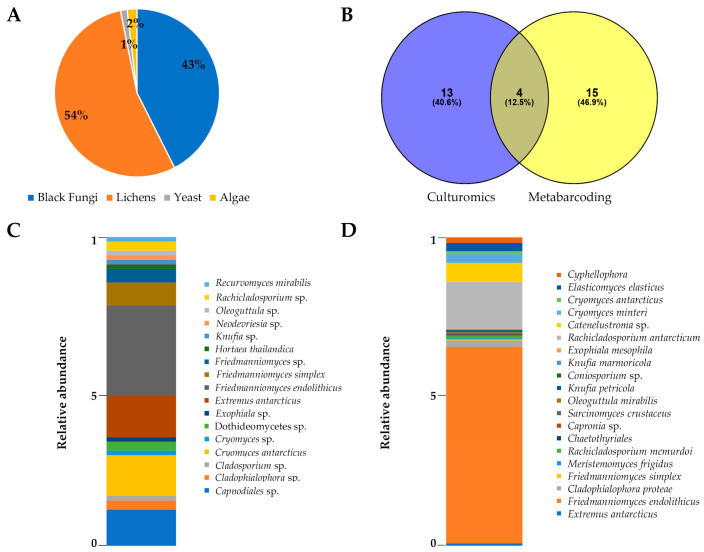
(**A**) The pie chart indicates the relative abundance of eukaryotes isolated from the Antarctic endolithic communities analyzed in this study. (**B**) Venn diagram reporting the distribution of black fungi retrieved from both culture-dependent and high-throughput sequencing (HTS) approaches. Percentages of both shared and unique operational taxonomic units (OTUs) are shown in parentheses. (**C**,**D**) Relative abundances of black fungi isolated (**C**) and recorded from Illumina amplicon sequencing raw data (**D**).

For each sample, the colonies were counted based on their morphology (Figure 2), resulting in a total of 4618 colonies belonging to black fungi (43%), 5904 to lichens (54%), 142 (1%) to yeasts, and 208 (2%) to algae, as reported in Table S2 (Figure 3A). In particular, RIF were the most abundant in almost all sites, particularly in Finger Mountain north (FMN) and University Valley south (UVS), while they were totally absent in Trio Nunatak south (TNS), The Mitten (TM), Pudding Butte north (PBN), Thern Promontory (THP), and Timber Peak south (TPS) (Table S2).

In Figure 3B, the Venn diagram shows that a substantial fraction of black fungal species recorded were unique for either cultivation or HTS (40.6 and 46.9% of the total, respectively), while only 12.6% were found using both approaches. Specifically, four taxa (*Cryomyces antarcticus*, *Extremus antarcticus*, *Friedmanniomyces endolithicus*, and *F. simplex*) were shared between the two groups.

Sixty-eight black fungal species were isolated using the culture-dependent approach. The species were identified based on their ITS sequences and classified into *Dothideomycetes* spp. and *Capnodiales* spp. at class and order levels, respectively (Table S3). The most abundant black fungi belonged to the genera *Friedmanniomyces* (40%), *Cryomyces* (14%), *Extremus* (13%), *Cladophialophora* (3%), and *Rachicladosporium* (3%). Conversely, the genera *Exophiala*, *Hortaea*, *Recurvomyces*, *Neodevriesia*, *Oleoguttula*, *Knufia*, and *Cladosporium* were found at lower percentages (<2%) (Table S3). The most frequent and abundant species isolated was *F. endolithicus* (29%), followed by *C. antarcticus* (13%) and *E. antarcticus* (13%), while the least frequent species were *H. thailandica* and *R. mirabilis* (<1% along the sampled area) (Figure 3C).

For the assessment of the community structure by HTS, DNA concentration was undetectable and PCR failure prevented the amplicon sequencing for samples collected at TNS, TM, Linnaeus Terrace south (LTS), Richard Nunatak (RN), TPS, and Mt. New Zealand south (MZS). 

The final ITS amplicon sequencing dataset contained 1,439,745 sequence reads across the dataset. Clustering was performed with UPARSE at 97% identity threshold, resulting in a total of 334 OTUs, for a total of 251 OTUs after the removal of singleton and rare taxa (<5 reads), as recently reported by Coleine et al. [22,23].

Nineteen OTUs (ca. 10% of all OTUs retrieved) were identified as black fungi (Table S4). Species identified were *E. antarcticus*, *F. endolithicus*, *F. simplex*, *Cladophialophora proteae*, *Meristemomyces frigidus*, *Rachicladosporium mcmurdoi*, *Sarcinomyces crustaceus*, *Oleoguttula mirabilis*, *Knufia petricola*, *K. marmoricola*, *Exophiala mesophila*, *Rachicladosporium antarcticum*, *Cryomyces minteri*, *C. antarcticus*, and *Elasticomyces elasticus*. Four OTUs were unclassified at the species level (*Capronia* sp., *Coniosporium* sp., *Catenelustroma* sp., *Cyphellophora* sp.) and one (OTU 183) at the family rank. According to cultivation data, *F. endolithicus* was the most abundant species, followed by *Catenelustroma* sp. and *R. antarcticum* (Figure 3D), not previously retrieved from cultivation analysis.

In Figure S1, the distribution and relative abundance of each species isolated is reported for each site, showing high heterogeneity across the sampled area. For instance, *F. endolithicus* was particularly abundant in Trio Nunatak north (TNN) and Richer Hills nort (RHN) and represented the only species isolated in LTS; MZS samples were dominated by *Cladophialophora* sp. and *Cladosporium* sp.; PBS, where six species (Capnodiales sp., *Exophiala* sp., *F. endolithicus*, *F. simplex*, *Knufia* sp., and *Neodevriesia* sp.) were isolated, showed the highest biodiversity.

In contrast, we observed homogeneity in black fungal community composition in samples analyzed by amplicon sequencing. *F. endolithicus* was highly predominant in almost all sites, except for Battleship Promontory, showing a higher diversity (Figure S2). *C. minteri* was also present in almost all sites but at lower percentage, while *E. elasticus* was present only in a few sites of the McMurdo Dry Valleys and at low frequency (Battleship Promontory (BP) north and south, LTN, and Knobhead south (KNS). Differently from culture-dependent results, *C. antarcticus* was retrieved from a few sites only and at the lowest percentages.

**Figure 4:**
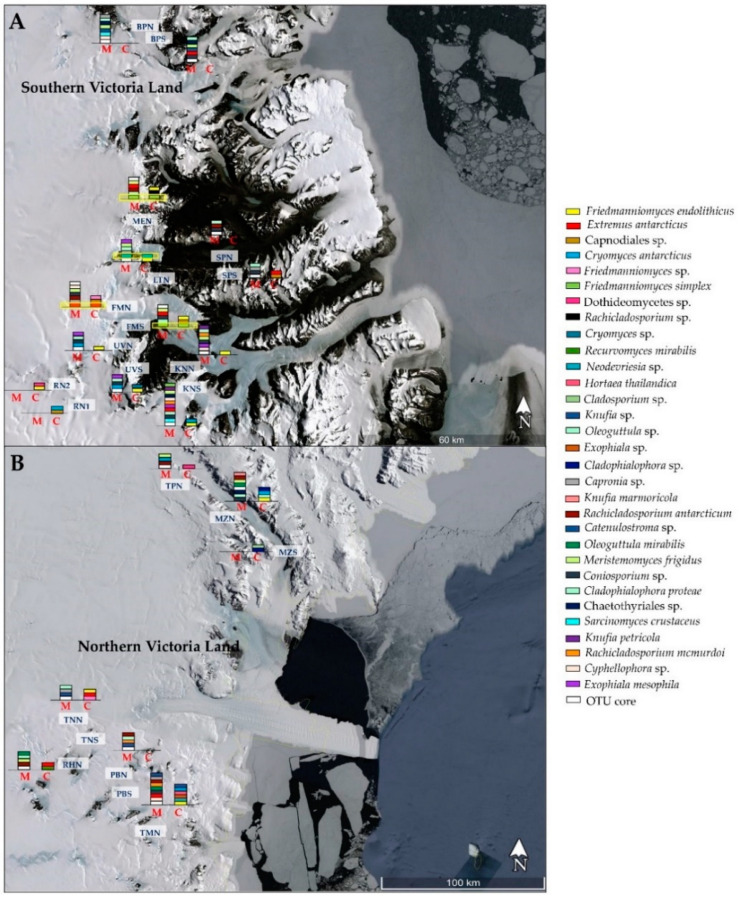
Map of distribution of black fungi along the Southern (**A**) and Northern (**B**) Victoria Land. Sites correspondences are reported in Table S1. The presence of each black fungal species retrieved in this study is reported: M—metabarcoding; C—culture-dependent approach.

Figure 4 represents a snapshot of RIF diversity along the Victoria Land, comparing cultivation and amplicon sequencing data. The distribution of fungi was highly heterogeneous; a few species were detected with both approaches; in particular, *F. simplex* in Mt. Elektra north (MEN) and Finger Mountain south (FMS), and *Neodevriesia* sp. and *E. antarcticus* in LTN and FMN, respectively.

### 3.2. Effect of Sun Exposure

The Upset diagram in Figure S3, built from the culture-dependent approach, indicates high heterogeneity across the samples. In particular, four species (*Neodevriesia* sp., *Knufia* sp., *H. thailandica*, and *Exophiala* sp.) form the “core” group and were present at all sites, as well as *F. endolithicus*, that was isolated from almost all sampled localities, and *C. antarcticus* and *E. antarcticus* were present in five sites in total. Conversely, several species such as *R. mirabilis*, *Rachicladosporium* sp., *Oleoguttula* sp., and *Cladophialophora* sp. were detected in one site only (Richer Hills, Mt. Elektra, and Mt. New Zealand, respectively).

Instead, the Upset diagram, generated from amplicon sequencing data, reported that the “core” is composed of *F. endolithicus*, *C. minteri*, and *E. elasticus*, that were present in all samples; on the other hand, several species such as *Catenelustroma* sp., *S. crustaceus*, *C. antarcticus*, *Cyphellophora* sp., and *E. mesophila* were less frequent (Figure S4).

**Figure 5:**
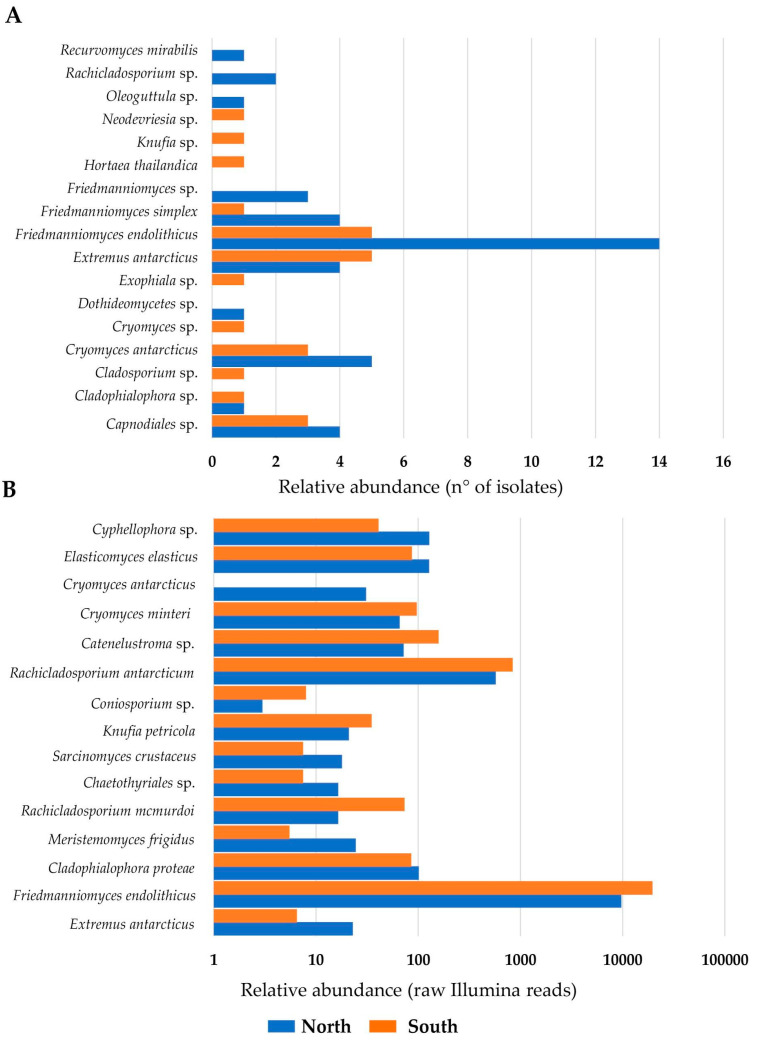
Presence/absence and relative abundance of each black fungal species recorded in this study by using a culture-dependent approach (**A**) or metabarcoding (**B**).

When we analyzed isolation data, we found that *F. endolithicus*, *C. antarcticus*, and a black unidentified fungus belonging to the order Capnodiales were more abundant in north sun-exposed rocks, while other taxa such as *Knufia* sp., *H. thailandica*, *Neodevriesia* sp., and *Exophiala* sp. were isolated from southern communities exclusively (Figure 5A). In comparison, using an amplicon sequencing approach (Figure 5B), numerous taxa (i.e., *F. endolithicus*, *C. minteri*, *Catenulostroma* sp., *R. antarcticum*, *R. mcmurdoi*, *Coniosporium* sp., and *K. petricola*) were more present in south-exposed rocks, where *C. antarcticus* was totally absent. Northern endolithic communities were dominated by *Cyphellophora* sp., *E. elasticus*, *E. antarcticus*, and *C. proteae*.

**Figure 6:**
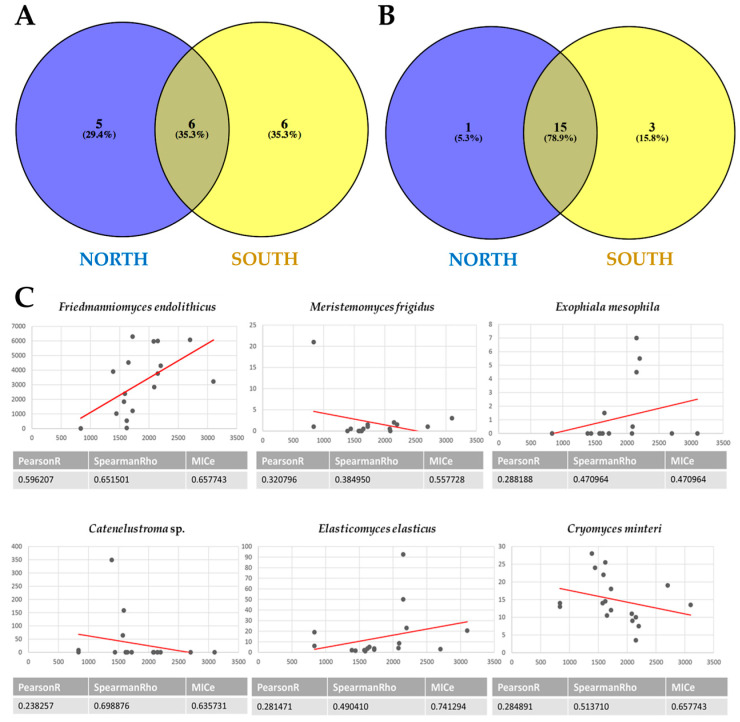
(**A**,**B**) Venn diagrams showing the distribution of black fungi between northern and southern exposure by using both a culture-dependent approach (**A**) and metabarcoding (**B**). Both the percentages of OTUs that were shared and found exclusively in each sun exposure are indicated. (**C**) Correlation analysis between species richness and altitude, calculated using MICtools v1.1.4; only rock-inhabiting fungi (RIF) that correlate with altitude (*p* < 0.05) are reported.

From cultivation data, the Venn diagram (Figure 6A) showed that the number of species shared between the north- and south-exposed samples and those unique for the two exposures is balanced (about 30%). In particular, the species *Capnodiales* sp., *Cladophialophora* sp., *C. antarcticus*, *E. antarcticus*, *F. endolithicus*, and *F. simplex* were shared. Conversely, *Dothideomycetes* sp., *Friedmanniomyces* sp., *Oleoguttula* sp., *Rachicladosporium* sp., and *R. mirabilis* were found exclusively in the north-exposed communities (29.4%), while six black fungal species (*Cladosporium* sp., *Cryomyces* sp., *Exophiala* sp., *H. thailandica*, *Knufia* sp., and *Neodevriesia* sp.) were unique for south-exposed communities.

Instead, Figure 6B indicates that a substantial fraction of OTUs (78.9%) were shared between the two sun exposures when we used HTS data. One species (*K. marmoricola*) was unique in the north-exposed surfaces, while *O. mirabilis*, *E. mesophila*, *Capronia* sp., and *F. simplex* (representing 15.8% of all RIF recorded) were unique southern-exposed communities.

### 3.3. Effects of Altitude on Black Fungi Relative Abundance

The results showed that the majority of black fungi did not correlate with altitude. A positive correlation (R > 0.90, *p* < 0.05) was found for *F. endolithicus* only. *E. mesophila* and *E. elasticus* did not show a positive correlation with altitude, but at 2000 m a.s.l., they reached an optimal condition reporting the highest abundance, while the relative abundance of *C. minteri* decreased according to the elevation.

## 4. Discussion

Cryptoendolithic microbial communities are self-sustaining ecosystems thriving in the most adverse conditions known on Earth, including the Antarctic ice-free areas where the limits for supporting life are approached. The absolute absence of higher plants and animals makes these ecosystems ideal model systems of low complexity to study the evolution of life in the extremes. Moreover, untangling biodiversity variation and composition under different degrees of environmental pressure may give clues to determine how microbes will adapt to environmental degradation, in an era of global warming, towards the dry limit for life.

Melanized rock fungi are poly-extreme tolerant microorganisms and recurrent components of Antarctic cryptoendolithic communities playing a primary role in the protection of the whole community, forming a black “sunscreen” barrier just above the photobiont stratification. For almost two decades, traditional culture-dependent approaches have been the most common methods applied to study black fungi of the Antarctic endolithic microbiome. Indeed, many taxa have been cultured, and a number of new genera and species have been described and continue to be found, while the distribution and species richness has not yet been thoroughly investigated. Despite the advantages of cultivation, including low cost, it provides a rather restricted view of microbial diversity and community structure in environmental samples as a consequence of the low percentage of microbes culturable in the lab. Similar conclusions have been recently reported by Zhang et al. [35] in a study that gave insights into deep-sea sediment fungal diversity targeted environmental DNA sequencing combined with traditional cultivation, and by Shinohara et al. [36] in a study that compared sequencing of fungal ITS2 with culture-dependent morphological identification to characterize house dust-borne fungal communities.

Besides, the next-generation sequencing technique has been successfully applied and has led to a deepened knowledge of fungal communities, including Antarctic endolithic mycobiota [21,22,23]. On the other hand, the short ITS reads gained with Illumina (~300 bp) do not provide adequate information for taxonomic assignment compared to complete ITS sequences (roughly 600 bp). We may, therefore, conclude that both approaches offer advantages and disadvantages that may be conveniently complemented.

The main purpose of this study was, thus, to provide the first “snapshot” of Antarctic cryptoendolithic black fungi in the ice-free areas of Victoria Land, evaluating a combination of culture-dependent and ITS1 sequencing. We analyzed samples spanning the Victoria Land in continental Antarctica and under increasing environmental pressure along an altitudinal gradient from 910 to 3100 m a.s.l. and differently sun-exposed rock surfaces.

With the culture-dependent approach, we were able to isolate RIF, lichenized fungi, yeasts, and microalgae. Despite the challenges in cultivating lichen-forming fungi under laboratory conditions, they represented the majority (54%), followed by RIF (43%), while yeasts and algae were reported at the lowest percentages (2% and 1%, respectively). Lichen symbioses represent the most successful obligate symbiotic association that promotes their success in the harshest environments on Earth [37]. They are extraordinarily adapted to the lithobiontic lifestyle and are predominant in the Antarctic lichen-dominated cryptoendolithic [2,6,21] and chasmoendolithic [38] communities, where the photobiont is primarily represented by the green alga *Trebouxia* (Chlorococcales) [39].

The determinant role of black fungi in these communities is confirmed by their high recurrency; they were isolated from rock specimens of all localities and with a high frequency, comparable to lichens; exceptions were for TNS, TM, THP, and TPS, where no black fungal colonies were obtained, but on the other hand, no other organism was isolated either (Table S2). Differently, from PBN, black fungi were not isolated, while a rather huge number of yeast colonies were counted. This evidence needs to be further verified; in fact, yeasts, as a rule, have been isolated at low frequency also in previous studies, having a side role as consumers together with other heterotrophs; they are commonly represented by cold-adapted basidiomycetous species belonging to the genera *Cryptococcus*, *Naganisha* (orders Filobasidiales and Tremellales), and *Rhodotorula* (order Cystobasidiales) [40,41].

On the other hand, combining the results of both cultivation and HTS, 32 black fungal taxa were found, among which 13 (40.6%) were specific to the former dataset, 15 (46.9%) to the Illumina data, and only 4 (12.5%) (*C. antarcticus*, *E. antarcticus*, *F. simplex*, and *F. endolithicus*) were shared in both datasets. Despite the higher number of species detected with the culture-independent approach in almost all samples analyzed, in many cases, the culture-dependent technique allowed to detect black fungal species that were overlooked otherwise. For instance, using the HTS, we were not able to detect RIF from RN and MZS, where DNA concentration was undetectable and amplification failed as well; conversely, it was possible to isolate six black fungal species from the same localities.

The evidence that species undetectable with the molecular approach could be successfully isolated by cultivation denotes the appropriate and essential complementarity of these two techniques. The failure of ITS1 sequencing to amplify all cultured fungi may be explained by the biases due to DNA extraction and PCR amplification in amplicon-based methods. Black fungi are, in fact, characterized by the presence of a thick and highly melanized cell wall that may hamper the efficiency of DNA extraction; this may also explain why *C. antarcticus*, having a particularly concrete and hard cell wall with additional melanized incrustations, is here much more easily detected with the culture-dependent method, while *C. minteri*, with an evidently lighter cell wall, was recorded from all localities, even if usually rarely isolated (Figure S4). Such a conspicuous cell wall confers to *C. antarcticus* a stunning resistance to a wide range of stressors, and it has been proposed as one of the few eukaryotic test organisms for astrobiological experiments. For instance, this species showed a high tolerance to accelerated He ions (150 MeV/n, LET 2.2 keV/µm) up to 1 kGy, γ-radiation (60Co) up to 55.61 kGy, and iron ions up to 1000 Gy in desiccated conditions [42,43]; it also survived 18 months of space exposure and simulated Martian conditions [19]. Further characterization of molecular adaptation and mechanisms of survival of this hyper tolerant RIF will be essential to guide our search for life elsewhere and define the limit of life, as we know it, on Earth.

The divergence between the two approaches at both species and genera level may be addressed to the small size of the Illumina reads compared to the whole ITS, as mentioned above. Among the 17 isolates, six were identified at the species level, as for *R. mirabilis* and *H. thailandica*, black fungi with a worldwide distribution [7,15,16]. The whole genome of *H. thailandica* (strain CCFEE 6315) was recently sequenced (genome size = 23.89 Mbp), showing that the majority of annotated genes are involved in basic cellular functions such as oxidoreductase activity and repair after UV irradiation [44]. Eleven taxa were not assigned to a known species, or in a few cases, to a known order, confirming that the Antarctic endolithic communities represent a paramount reservoir of new taxa.

Conversely, the endemic *R. mcmurdoi* was retrieved exclusively from the ITS1 dataset together with other species that are globally distributed in rocky environments, such as *K. marmoricola* and *C. protaea*, or *E. elasticus*, that has particular success in cold, dry environments. Instead, members of the genus *Exophiala* have been found using both approaches. Within the Herpotrichiellaceae family (Chaetothyriales), *Exophiala* spp. are adapted to a wide range of ecological niches, including human environments such as swimming pools and bathing facilities [45,46]. Several species have been isolated from glaciers [47] and other extremely cold habitats, including Antarctic endolithic communities [48].

*F. endolithicus* was the most abundant and frequent species recorded with both approaches and the only species isolated in some of the harshest locations, as for LTS. The high frequency and spread of this species even at altitudes above 3300 m a.s.l. was previously observed [6,21], suggesting a high degree of adaptation and specialization to the prohibitive conditions of such environments. To date, the endemic genus *Friedmanniomyces* includes two described species of rock-inhabiting meristematic black fungi, *F. simplex* and *F. endolithicus* [7]. Recently, the whole-genome sequencing of these two species revealed enriched genomic traits for response to salt, X-rays, cold, and DNA damage, while other genomic features such as meristematic growth and cold adaptation were found exclusively in *F. endolithicus*, confirming its abilities to survive a wide variety of stresses [44].

A notable variability across the sampled area in presence/absence and relative abundance has been observed in cultivation data. For instance, *F. endolithicus* was particularly abundant in a few sites (Trio Nunatak and Richer Hills north). Other species were rare and present in one site only; e.g., *Neodevriesia* sp., *Knufia* sp., *Exophiala* sp., and *H. thailandica* were found at PBS exclusively.

The huge heterogeneity in biodiversity and structure in Antarctic endolithic communities collected in localities with similar environmental conditions have been already reported by previous studies [6,21]. Besides, a high degree of variability has been demonstrated even in rocks collected in strict proximity to each other in the same site both in the Northern and Southern Victoria Land, supporting the hypothesis that this variability may not be related to the breadth of the sampling area, but rather to the homogeneity of the substratum and the microclimatic parameters [49,50]. In contrast, we observed a higher homogeneity in black fungal community composition in samples analyzed by amplicon sequencing, but it was mainly due to the massive predominance of *F. endolithicus* in all sites, except for BP, which showed a higher diversity (Figure S2).

We also tested the effect of sun exposure on the distribution and diversity of black fungi and compared the two approaches. The influence of this environmental parameter has already been reported in shaping the diversity and response of the whole mycobiota [22,51]. In these studies, an increased relative abundance of RIF has been observed in the shaded south-exposed rocks, which are prone to stronger environmental constraints. This is explained by the peculiar ecology of these fungi that are able to survive low temperatures and drought and to successfully spread where other microorganisms are hampered by environmental constraints [8]. In particular, *F. endolithicus* was found as the most recurrent RIF in the communities of the shady rocks and accounted as a marker species for the harshest conditions [23]. In the present study, we found that this environmental stress greatly influences black fungi, particularly when considering culture-dependent data. Indeed, 35% of taxa were shared among north and south exposures, while an important percentage of unique taxa were reported in north and south communities (30% and 35%, respectively). Looking at the HTS dataset, instead, almost all taxa (78%) were shared, while only a few taxa were unique to northern and southern surfaces (one and three taxa, respectively). Cultivation data indicated also that *E. antarcticus* was the only one predominant in south-exposed surfaces, while all other species were more abundant in the opposite sun-exposed samples. Conversely, seven species were more abundant in south-exposed rocks in HTS, and *F. endolithicus* was confirmed as the marker species for the harshest conditions, while it resulted as being almost three times more abundant in north-exposed rocks when analyzed by the culture-dependent approach. These apparently contrasting results may be a consequence of the higher resolution of HTS, leading to a more reliable interpretation of the community organization in many cases.

The influence of elevation in Antarctic endolithic communities have been recently studied by analyzing changes in both bacterial and fungal species richness and their relative abundance [21,52], while the analysis of patterns displayed by black fungal diversity and elevation have received little attention. Therefore, in the present study, we also aimed to investigate whether RIF were correlated with altitude and increasing environmental pressure and to determine an eventual elevational diversity gradient (EDG), where diversity changed with elevation. We found that most species did not correlate with the altitude, while a significant correlation was reported for six species only (i.e., *E. elasticus*, *Catenulostroma* sp., *E. mesophila*, *C. minteri*, *M. frigidus*, and *F. endolithicus*). For the first four species, the relative abundance increased steeply up to a certain point (1500–2000 m a.s.l.), creating a “diversity bulge” at middle elevations; above this altitude, a decrease was evident. Conversely, we reported that *F. endolithicus* tended to increase with elevation, confirming this taxon as highly tolerant and adapted to the most extreme conditions. These findings support the hypothesis that the increasing environmental pressure may have led to the selection of the most tolerant and adapted species at the highest altitude.

## 5. Conclusions

In this work, the combination of fungal ITS1 high-throughput sequencing and a culture-dependent approach provided a clearer and more detailed picture of the black fungal diversity and abundance in the Antarctic cryptoendolithic communities in the ice free-areas of continental Antarctica. Both methods applied showed advantages and disadvantages; for instance, the use of amplicon sequencing captured the diversity of environmental microbiota in deeper detail, but several black fungi that were successfully isolated were not detected by this approach. Limitations also emerged with classic culture techniques for fungal isolation. In particular, we found only a 12% overlap of species detected when both approaches were applied. The results obtained in this study clearly sustain that the application of both approaches is desirable to discern the biodiversity in deeper detail, since the respective limits are complementary. Culture-dependent studies are insufficient for a fine study of the effect of environmental parameters, as they lack the resolution needed to distinguish community composition but do complement these high-throughput studies with the chance to visualize, test the functions, and explore the genetics and genomics of these extremophiles.

## Data Availability

All raw sequence data have been submitted to the GenBank databases under BioProject accession numbers PRJNA379160, PRJNA453198 and PRJNA379160.

## Supplementary Materials

The following are available online at https://www.mdpi.com/2309-608X/7/3/213/s1, Figure S1: Relative abundance of black fungi isolated from this study are reported for each site. Sites correspondence are reported in Table S1. Figure S2: Relative abundance of black fungi retrieved from Illumina amplicon sequencing raw data are reported for each site. Sites correspondence are reported in Table S1. Figure S3: Number of the shared black fungal species (intersections size), recorded from culture-dependent approach, among the sampled sites. Sites correspondence are reported in Table S1. Figure S4: Number of the shared black fungal species (intersections size), recorded from Illumina amplicon sequencing, among the sampled sites. Sites correspondence are reported in Table S1. Table S1: Metadata of visited sites in the Victoria Land (continental Antarctica) during the XXXI Italian Antarctic Expedition (December 2015–January 2016) include: altitude, air temperature, sun exposure, relative humidity and geographic coordinates. Sites correspondence is also reported. Table S2: Presence and relative abundance (colony counts) of culturable eukaryotic strains are reported for each site. Sites correspondence is reported in Table S1. Table S3: Taxonomic identification, based on Internal Transcribed Spacer (ITS) region compared to Genbank database, of black fungi analyzed in this study. Table S4: List of black fungal Operational Taxonomic Units (OTUs) identified at 97% identity from Illumina amplicon sequencing.
