# Supplementary material for: Culture-Dependent and Amplicon Sequencing Approaches Reveal Diversity and Distribution of Black Fungi in Antarctic Cryptoendolithic Communities
Source: J Fungi (Basel). 2021 Mar 16;7(3):213. doi: 10.3390/jof7030213 (PMC8001563; doi:10.3390/jof7030213)
Supplement: Supplementary file 1 [file jof-07-00213-s001.zip › Table S2.docx]

| **Site** | **Black Fungi** | **Lichenized fungi** | **Yeasts** | **Algae** |
| --- | --- | --- | --- | --- |
| BPN | 100 | 150 | 1 | 100 |
| BPS | 100 | 50 | - | - |
| TNN | 22 | - | 69 | - |
| TNS | - | - | - | - |
| RHN | 6 | 36 | 4 | - |
| TM | - | - | - | - |
| SPN | 371 | 893 | - | - |
| SPS | 335 | 110 | - | - |
| LTN | 270 | 139 | - | - |
| RN1 | 3 | 25 | - | - |
| PBN | - | - | 50 | - |
| PBS | 90 | 20 | 15 | - |
| FMN | 1097 | 749 | - | 33 |
| FMS | 83 | 876 | - | 24 |
| RN2 | 2 | 68 | - | - |
| LTS | 1 | - | - | - |
| THP | - | - | - | - |
| MEN | 278 | 290 | - | 51 |
| UVN | 341 | 1440 | - | - |
| KN | 4 | 203 | - | - |
| KS | 13 | 1 | - | - |
| UVS | 1270 | 755 | - | - |
| TPN | 12 | 29 | - | - |
| TPS | - | - | - | - |
| MZN | 130 | 70 | - | - |
| MZS | 90 | - | 3 | - |
