# Supplementary material for: Culture-Dependent and Amplicon Sequencing Approaches Reveal Diversity and Distribution of Black Fungi in Antarctic Cryptoendolithic Communities
Source: J Fungi (Basel). 2021 Mar 16;7(3):213. doi: 10.3390/jof7030213 (PMC8001563; doi:10.3390/jof7030213)
Supplement: Supplementary file 1 [file jof-07-00213-s001.zip › Table S3.docx]

| **Taxonomic identification** | **CCFEE** | **Identity (%)** |
| --- | --- | --- |
| *Capnodiales* sp. | 6656 | 90.46 |
| *Capnodiales* sp. | 6471 | 87.56 |
| *Capnodiales* sp. | 6472 | 90.59 |
| *Capnodiales* sp. | 6473 | 90.04 |
| *Capnodiales* sp. | 6595 | 90.66 |
| *Capnodiales* sp. | 6654 | 90.53 |
| *Capnodiales* sp. | 6655 | 89.88 |
| *Capnodiales* sp. | 6682 | 90.50 |
| *Cladophialophora* sp. | 6417 | 92.51 |
| *Cladophialophora* sp. | 6465 | 91.35 |
| *Cladosporium* sp. | 6418 | 100 |
| *Cryomyces antarcticus* | 6505 | 99.43 |
| *Cryomyces antarcticus* | 6650 | 100 |
| *Cryomyces antarcticus* | 6513 | 97.05 |
| *Cryomyces antarcticus* | 6459 | 99.79 |
| *Cryomyces antarcticus* | 6414 | 99.79 |
| *Cryomyces antarcticus* | 6463 | 99.76 |
| *Cryomyces antarcticus* | 6468 | 99.80 |
| *Cryomyces antarcticus* | 6467 | 100.00 |
| *Cryomyces antarcticus* | 6469 | 99.25 |
| *Cryomyces* sp. | 6591 | 99.44 |
| *Dothideomycetes* sp. CCFEE 502 | 6484 | 98.09 |
| *Dothideomycetes* sp. CCFEE 502 | 6508 | 99.36 |
| *Exophiala* sp. | 6462 | 100 |
| *Extremus antarcticus* | 6515 | 100 |
| *Extremus antarcticus* | 6479 | 100 |
| *Extremus antarcticus* | 6596 | 100 |
| *Extremus antarcticus* | 6681 | 100 |
| *Extremus antarcticus* | 6522 | 100 |
| *Extremus antarcticus* | 6477 | 100 |
| *Extremus antarcticus* | 6474 | 100 |
| *Extremus antarcticus* | 6475 | 100 |
| *Extremus antarcticus* | 6523 | 99.57 |
| *Friedmanniomyces endolithicus* | 6517 | 98.45 |
| *Friedmanniomyces endolithicus* | 6631 | 98.45 |
| *Friedmanniomyces endolithicus* | 6507 | 98.52 |
| *Friedmanniomyces endolithicus* | 6520 | 98.81 |
| *Friedmanniomyces endolithicus* | 6651 | 99.80 |
| *Friedmanniomyces endolithicus* | 6633 | 99.61 |
| *Friedmanniomyces endolithicus* | 6524 | 100.00 |
| *Friedmanniomyces endolithicus* | 6635 | 99.80 |
| *Friedmanniomyces endolithicus* | 6653 | 100 |
| *Friedmanniomyces endolithicus* | 6481 | 100 |
| *Friedmanniomyces endolithicus* | 6521 | 100 |
| *Friedmanniomyces endolithicus* | 6506 | 100 |
| *Friedmanniomyces endolithicus* | 6629 | 99.80 |
| *Friedmanniomyces endolithicus* | 6589 | 100 |
| *Friedmanniomyces endolithicus* | 6416 | 99.80 |
| *Friedmanniomyces endolithicus* | 6464 | 99.79 |
| *Friedmanniomyces endolithicus* | 6466 | 99.61 |
| *Friedmanniomyces endolithicus* | 6470 | 99.79 |
| *Friedmanniomyces endolithicus* | 6458 | 99.60 |
| *Friedmanniomyces endolithicus* | 6588 | 99.79 |
| *Friedmanniomyces simplex* | 6511 | 94.88 |
| *Friedmanniomyces simplex* | 6478 | 97.12 |
| *Friedmanniomyces simplex* | 6485 | 99.15 |
| *Friedmanniomyces simplex* | 6483 | 97.44 |
| *Friedmanniomyces simplex* | 6652 | 100 |
| *Friedmanniomyces* sp. | 6680 | 98.45 |
| *Friedmanniomyces* sp. | 6482 | 94.97 |
| *Friedmanniomyces* sp. | 6612 | 90.13 |
| *Hortaea thailandica* | 6415 | 99.61 |
| *Knufia* sp. | 6461 | 94.80 |
| *Neodevriesia* sp. | 6460 | 93.93 |
| *Oleoguttula* sp. | 6420 | 97.57 |
| *Rachicladosporium* sp. | 6480 | 95.68 |
| *Rachicladosporium* sp. | 6514 | 93.36 |
| *Recurvomyces mirabilis* | 6590 | 99.20 |
