# Supplementary material for: Culture-Dependent and Amplicon Sequencing Approaches Reveal Diversity and Distribution of Black Fungi in Antarctic Cryptoendolithic Communities
Source: J Fungi (Basel). 2021 Mar 16;7(3):213. doi: 10.3390/jof7030213 (PMC8001563; doi:10.3390/jof7030213)
Supplement: Supplementary file 1 [file jof-07-00213-s001.zip › Table S4.docx]

| **Correspondence** | **OTU ID** | **Taxonomy** |
| --- | --- | --- |
| 1 | OTU100 | *Extremus antarcticus* |
| 2 | OTU12 | *Friedmanniomyces endolithicus* |
| 3 | OTU130 | *Cladophialophora proteae* |
| 4 | OTU132 | *Friedmanniomyces simplex* |
| 5 | OTU135 | *Meristemomyces frigidus* |
| 6 | OTU147 | *Rachicladosporium mcmurdoi* |
| 7 | OTU183 | Chaetothyriales sp. |
| 8 | OTU188 | *Capronia* sp. |
| 9 | OTU189 | *Sarcinomyces crustaceus* |
| 10 | OTU201 | *Oleoguttula mirabilis* |
| 11 | OTU218 | *Knufia petricola* |
| 12 | OTU338 | *Coniosporium* sp. |
| 13 | OTU347 | *Knufia marmoricola* |
| 14 | OTU403 | *Exophiala mesophila* |
| 15 | OTU404 | *Rachicladosporium antarcticum* |
| 16 | OTU68 | *Catenelustroma* sp. |
| 17 | OTU93 | *Cryomyces minteri* |
| 18 | OTU94 | *Cryomyces antarcticus* |
| 19 | OTU96 | *Elasticomyces elasticus* |
| 20 | OTU99 | *Cyphellophora* sp. |
