# Supplementary material for: Culture-Dependent and Amplicon Sequencing Approaches Reveal Diversity and Distribution of Black Fungi in Antarctic Cryptoendolithic Communities
Source: J Fungi (Basel). 2021 Mar 16;7(3):213. doi: 10.3390/jof7030213 (PMC8001563; doi:10.3390/jof7030213)
Supplement: Supplementary file 1 [file jof-07-00213-s001.zip › Table S1.docx]

| **Site** | **Locality** | **Sun exposure** | **Altitude**  **(m a.s.l.)** | **Humidity**  **(%)** | **Temp. (°C)** | **Coordinates** |
| --- | --- | --- | --- | --- | --- | --- |
| BPN | Battleship Promontory | North | 910 | 22.9 | -4.4 | 76°54’04.0"S 160°54'36.6"E |
| BPS | Battleship Promontory | South | 910 | 22.9 | -4.4 | 76°54’06.3"S 160°54'38.9"E |
| TNN | Trio Nunatak | North | 1,388 | 40.9 | -5.1 | 75°28’56.6"S 159°35'28.3"E |
| TNS | Trio Nunatak | South | 1,388 | 40.9 | -5.1 | 75°28’56.6"S 159°35'28.3"E |
| RHN | Ricker Hills | North | 1,442 | 42.7 | -7.2 | 75°42’14.6"S 159°13'39.4"E |
| TM | The Mitten | - | 1,470 | - | - | 75°59′S 160°30′E |
| SPN | Siegfried Peak | North | 1,620 | 52.8 | -9.3 | 77°34’43.3"S 161°47'11.7"E |
| SPS | Siegfried Peak | South | 1,620 | 54.9 | -6.8 | 77°34’39.9"S 161°47'17.4"E |
| LTN | Linnaeus Terrace | North | 1,649 | 58.6 | -9.6 | 77°36’01.3"S 161°05'00.5"E |
| RN1 | Richard Nunatak site1 | - | 1,686 | - | - | 75°56’02.14"S 159°47'52.6"E |
| PBN | Pudding Butte | North | 1,690 | 32.4 | -8.5 | 75°51’30.2"S 159°58'25.7"E |
| PBS | Pudding Butte | South | 1,690 | NA | NA | 75°51’33.01"S 159°58'26.6"E |
| FMN | Finger Mt. | North | 1,720 | 35.1 | -6.4 | 77°45’0.93"S 160°44'45.2"E |
| FMS | Finger Mt. | South | 1,720 | 35.1 | -6.4 | 77°45’10"S 160°44'44.39.7"E |
| RN2 | Richard Nunatak site 2 | - | 1,721 | - | - | 77°56’0.6"S 159°48'01.1"E |
| LTS | Linnaeus Terrace | South | 1,761 | 68.2 | -12.6 | 77°37’09.9"S 161°11'50.8"E |
| THP | Thern Promontory | - | 1,810 | 53.4 | -15.8 | 74°34’43.4"S 162°14'28.3"E |
| MEN | Mt. Elektra | North | 2,080 | 63 | -11.9 | 77°29’28.0"S 160°54'16.4"E |
| UVN | University Valley | North | 2,090 | 18 | -14 | 77°52’28.6"S 160°44'22.6"E |
| KNN | Knobhead | North | 2,150 | 50 | -12.5 | 77°54’37.8"S 161°34'48.8"E |
| KNS | Knobhead | South | 2,150 | 38.9 | -8.9 | 77°54’43.6"S 161°34'39.3"E |
| UVS | University Valley | South | 2,200 | 39.1 | -11.2 | 77°52’21.5"S 160°45'19.2"E |
| TPN | Timber Peak | North | 2,702 | 30.1 | -12.4 | 74°10’10.5"S 162°25'38.0"E |
| TPS | Timber Peak | South | 2,702 | 30.1 | -12.4 | 74°10’10.5"S 162°25'38.0"E |
| MZN | Mt. New Zealand | North | 3,100 | 47.6 | -17.2 | 74°10’44.0"S 162°30'53.0"E |
| MZS | Mt. New Zealand | South | 3,100 | 47.6 | -17.2 | 74°10’44.0"S 162°30'53.0"E |
