# Supplementary figures and images for: Culture-Dependent and Amplicon Sequencing Approaches Reveal Diversity and Distribution of Black Fungi in Antarctic Cryptoendolithic Communities
Source: J Fungi (Basel). 2021 Mar 16;7(3):213. doi: 10.3390/jof7030213 (PMC8001563; doi:10.3390/jof7030213)

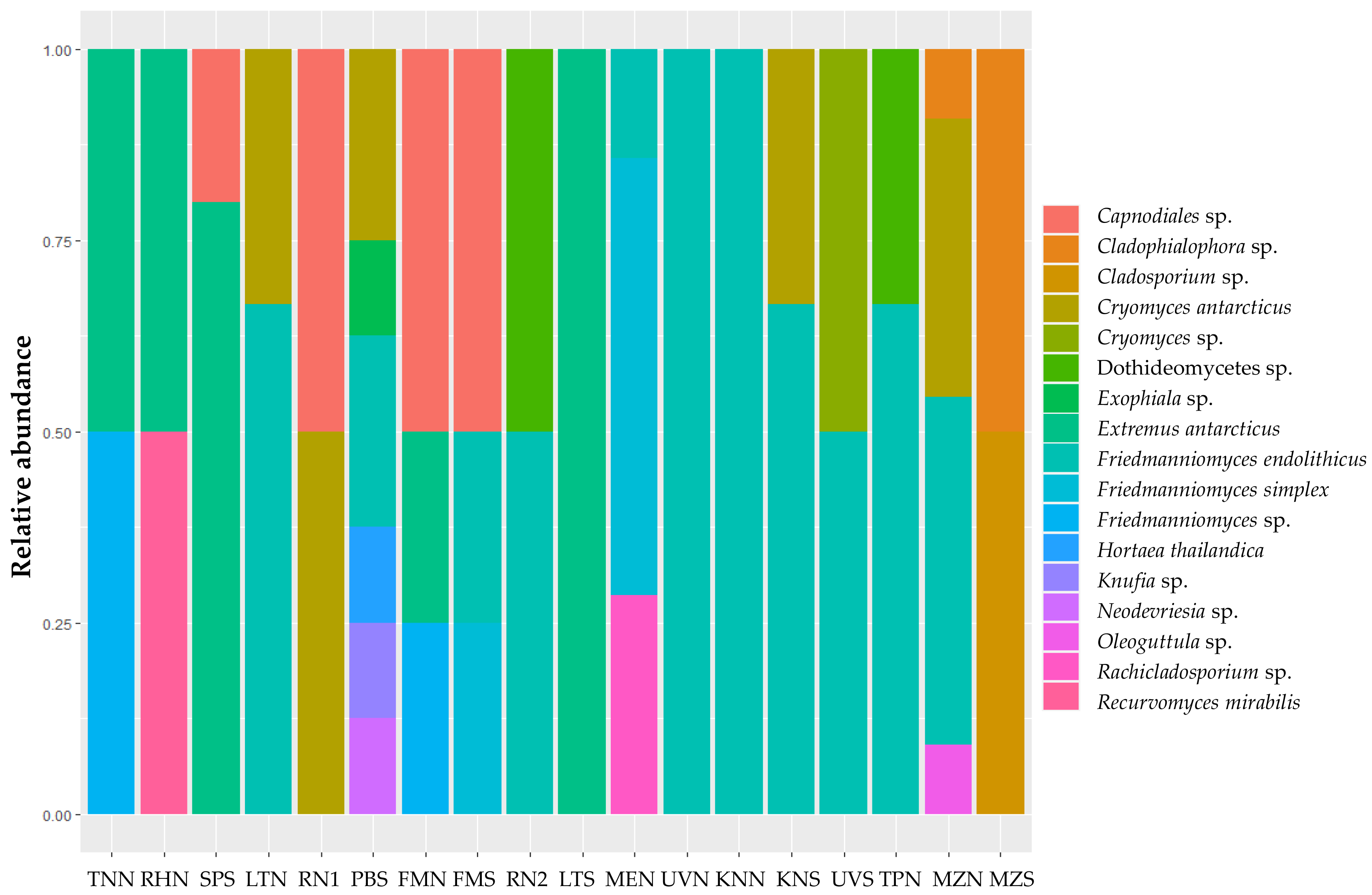

Supplement: Supplementary file 1 [file jof-07-00213-s001.zip › Figure S1.tif]

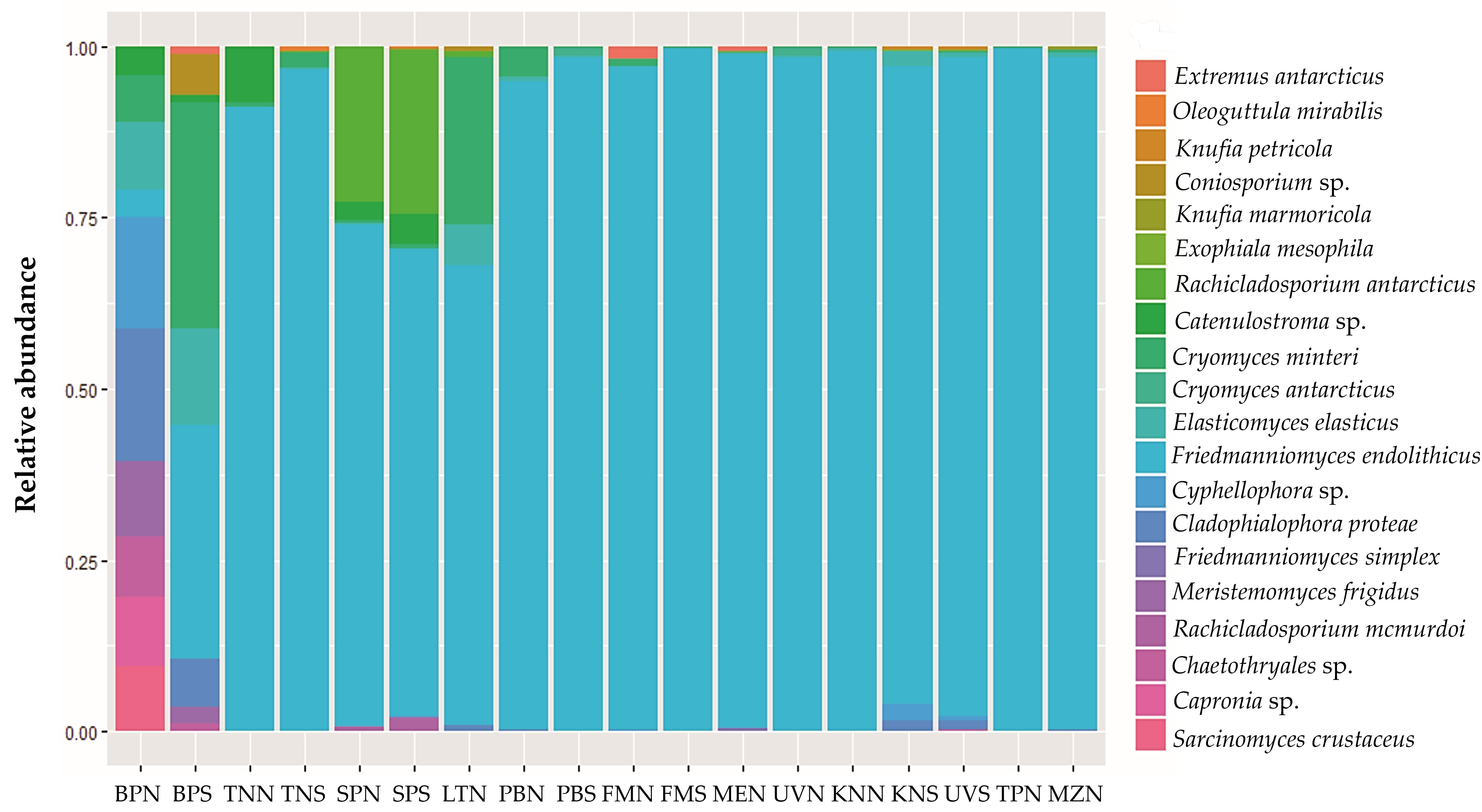

Supplement: Supplementary file 1 [file jof-07-00213-s001.zip › Figure S2.jpg]

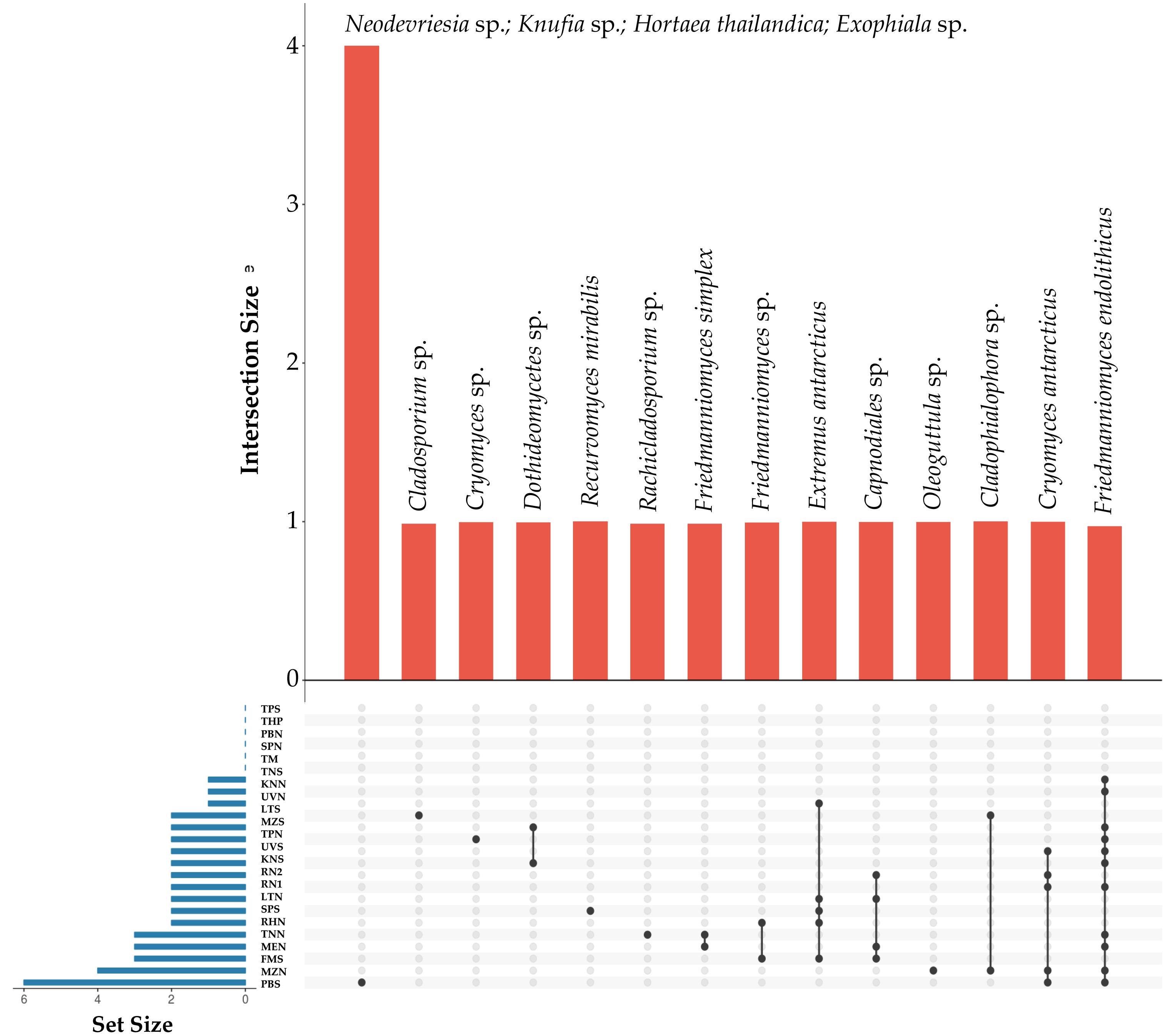

Supplement: Supplementary file 1 [file jof-07-00213-s001.zip › Figure S3.tif]

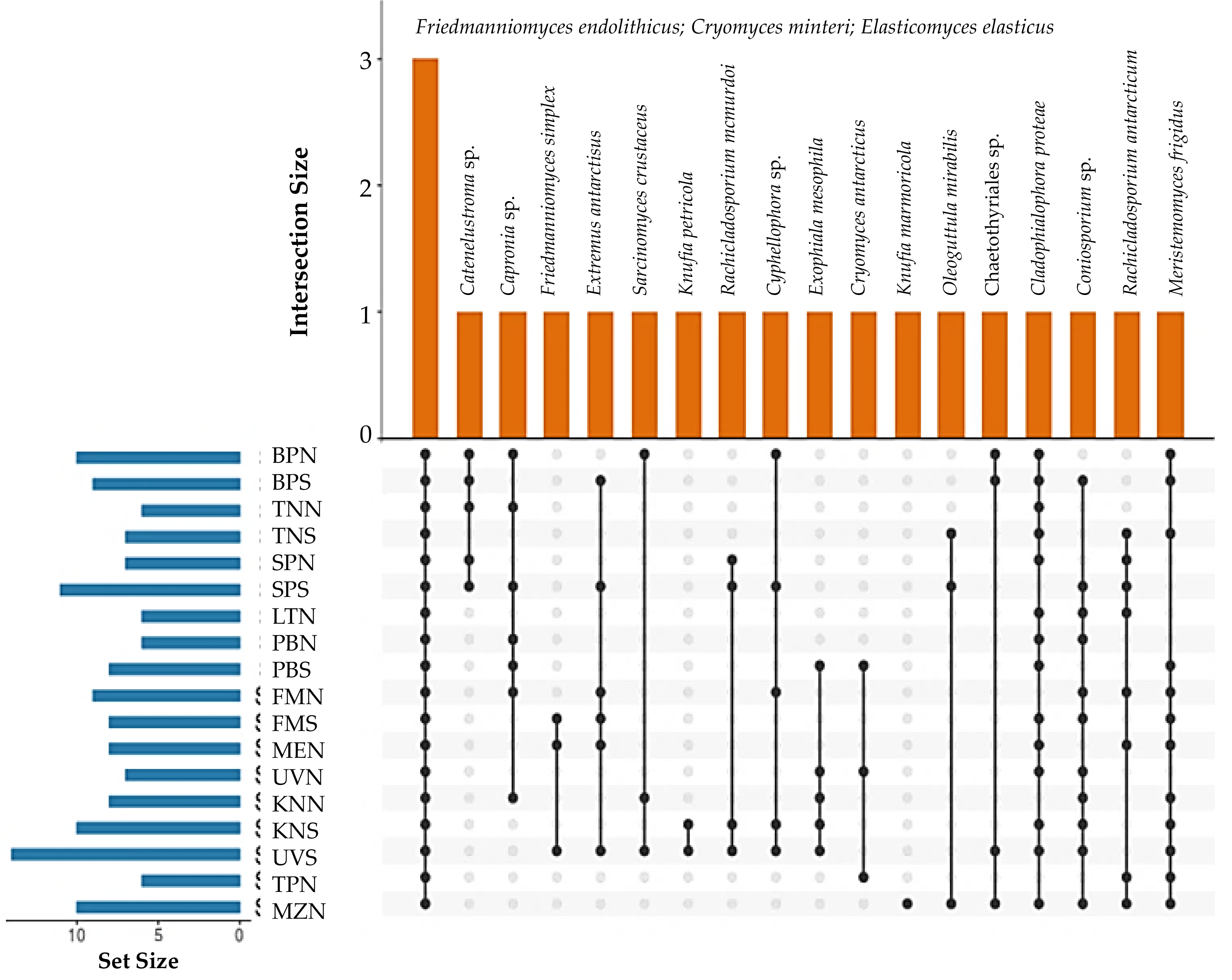

Supplement: Supplementary file 1 [file jof-07-00213-s001.zip › Figure S4.tif]
